# Activating Effect of Benzbromarone, a Uricosuric Drug, on Peroxisome Proliferator-Activated Receptors

**DOI:** 10.1155/2007/36092

**Published:** 2007-08-27

**Authors:** Chiyoko Kunishima, Ikuo Inoue, Toshihiro Oikawa, Hiromu Nakajima, Tsugikazu Komoda, Shigehiro Katayama

**Affiliations:** ^1^Department of Pharmacovigilance, Torii Pharmaceutical Co., Ltd., 3-4-1 Nihonbashi-honcho, Chuo-ku, Tokyo 103-8439, Japan; ^2^Department of Internal Medicine, Faculty of Medicine, Saitama Medical University, 38 Morohongo, Moroyama, Iruma-gun, Saitama 350-0495, Japan; ^3^Department of Clinical Laboratory, Osaka Medical Center for Cancer and Cardiovascular Diseases, 1-3-3 Nakamichi, Higashinari-ku, Osaka 537-8511, Japan; ^4^Department of Biochemistry, Faculty of Medicine, Saitama Medical University, 38 Morohongo, Moroyama, Iruma-gun, Saitama 350-0495, Japan

## Abstract

Benzbromarone, a uricosuric drug, reportedly causes hepatic hypertrophy accompanied by proliferation of peroxisomes in rats. To elucidate the mechanisms underlying induction of peroxisome proliferation by benzbromarone, we examined binding affinity for peroxisome proliferator-activated receptor α (${\text{PPAR}}_{\alpha }$) and γ (${\text{PPAR}}_{\gamma }$), and effects on the binding activity of PPARs with peroxisome proliferation-responsive element (PPRE) and expression of the PPARs target protein. Binding affinity of benzbromarone for ${\text{PPAR}}_{\alpha }$ and ${\text{PPAR}}_{\gamma }$ was examined by reporter gene assay. Binding activity of PPARs with PPRE was determined by electric mobility shift assay, and expression of lipoprotein lipase (LPL) and acyl-CoA synthetase (ACS) by Western blot method. Benzbromarone displayed affinity for ${\text{PPAR}}_{\alpha }$ and ${\text{PPAR}}_{\gamma }$, and promoted binding of PPARs to PPRE. Furthermore, cultured cells with benzbromarone added showed upregulated expression of LPL and ACS. These results suggest that benzbromarone induces peroxisome proliferation in hepatocytes by binding to PPARs, and controls expression of proteins related to lipid metabolism.

## 1. INTRODUCTION

Benzbromarone is a uricosuric drug that has been widely used for more than 25 years as a therapeutic agent for hyperuricemia. Recently, urate transporter 1 (URAT1) was identified in the luminal membrane of the proximal renal tubule, and the mechanism of action for benzbromarone was identified as suppression of uric acid resorption via inhibition of URAT1 [1]. Regarding the toxicity of benzbromarone, hypertrophy of the liver accompanied by proliferation of peroxisomes has been reported in a repeated-dose toxicity study in rats [2]. Similar changes have also been observed in studies on clofibrate and fenofibrate, both of which are antihyperlipemic drugs [2, 3]. Clofibrate and fenofibrate represent activators of peroxisome proliferator-activated receptor *α* (PPAR_*α*_), and benzbromarone may similarly cause hypertrophy of the liver via actions on PPAR_*α*_.

We have previously demonstrated that benzbromarone has affinity for PPAR_*α*_ and PPAR_*γ*_, and upregulates expression of each protein [4]. In this study, to clarify the actions of benzbromarone on PPARs, the affinity of benzbromarone for PPAR_*α*_ and PPAR_*γ*_ was reexamined in comparison with a representative activator of each receptor. In addition, regarding the mechanism of gene expression induced by benzbromarone, binding between PPARs and peroxisome proliferator-responsive element (PPRE), and protein expressions of lipoprotein lipase (LPL), and acyl-CoA synthetase (ACS) encoded by the target gene of PPAR were examined.

Based on the findings, hepatic hypertrophy due to benzbromarone and the influence of benzbromarone on lipid metabolism are discussed.

## 2. MATERIALS AND METHODS

### 2.1. Chemicals

Benzbromarone, an active pharmaceutical ingredient of Urinorm marketed by Torii Pharmaceutical Co., Ltd. (Tokyo, Japan), was used as a test compound in this study. Troglitazone, pioglitazone and fenofibric acid were synthesized at the Central Research Pharmaceutical Institute of Japan Tobacco Inc. (Osaka, Japan). Clofibric acid was purchased from Sigma-Aldrich Japan (Tokyo, Japan), nuclear and cytoplasmic extraction reagents from Pierce (Ill, USA), and a nucleic acid labeling kit from Molecular Probes (Ore, USA).

### 2.2. Cell culture

NIH/3T3 cells and human kidney 293T cells were maintained in D-MEM containing 10% fetal bovine serum, 50 U/mL penicillin and 50 *μ*g/mL streptomycin at 37°C in a humidified 95% air and 5% CO_2_ atmosphere.

### 2.3. Reporter gene analysis

NIH/3T3 cells ($0.4\times 10^{5}$ cells/well) were cultured in 24-well plates for 20 hours and then transfected with a receptor plasmid for the chimera of PPAR_*γ*_ or the PPAR_*α*_ ligand-binding domain and GAL4 DNA-binding domain, together with a receptor plasmid containing the GAL4-responsive promoter driving expression of luciferase. After 6 hours, cells were given fresh D-MEM containing test compounds at final concentrations of 0.001–100 *μ*M, and were cultured for an additional 2 days. Benzbromarone, clofibric acid, fenofibric acid, troglitazone, and pioglitazone were suspended in 0.1% DMSO solution. After cells had been lysed, luciferase activity was determined using a CT-9000D luminometer (Dia-iatron, Tokyo, Japan).

A total of 3 measurements were performed for each batch of cultured cells per test, and a total of 3 tests were performed.

### 2.4. Electric mobility shift assay (EMSA)

First, following addition of benzbromarone at a final concentration of 1 *μ*M, 293T cells were cultured for 24 hours. Cells were washed with TBS and pelleted by centrifugation at 1500×g for 5 minutes. Pellets were suspended in 1 mL TBS and transferred into an Eppendorf tube, then centrifuged again at 1000×g for 5 minutes to obtain pellets. Double-stranded oligonucleotides for sequences of the PPRE and mutant of PPRE were purchased from Santa Cruz Biotechnology (Calif, USA). Oligonucleotides were labeled with Alexa Flour 488 using a ULYSIS Nucleic Acid Labeling Kit (Molecular Probes). Labeled oligonucleotides (500 ng) were added to nuclear extracts. The binding assay buffer comprised 20 mM HEPES (pH 7.5) containing 40 mM KCl, 5% glycerol, and 1 *μ*g dl-dC. Assay was performed at room temperature for 10 minutes at a final volume of 25 *μ*L. Products of the binding reaction were separated on 5% polyacrylamide gel run in Tris-borate-EDTA at room temperature at 100 V for 60 minutes. Gels were visualized using a Fluorimager SI (Molecular Dynamics, Tokyo, Japan). This test was performed 6 times.

### 2.5. Western blot analysis

Benzbromarone was added to 293T cells to achieve a final concentration of 1 or 10 *μ*M, and cells were cultured for 24 hours. Western blots for LPL and ACS proteins were then performed according to established procedures. Briefly, after cells (10^8^ cells/well) had been suspended in lysis buffer (10 mM sodium phosphate, 150 mM NaCl, 0.5% sodium deoxycholate, 0.1% SDS, 100 *μ*g/mL PMSF, 30 *μ*g/mL aprotinin, and 1 mM sodium orthovanadate, pH 7.4), aliquots of the cell suspension were obtained by repetitive pipetting and lysed by incubation at 4°C for 15 minutes. Lysed cells were centrifuged at 400×g for 10 minutes, and the supernatant was separated by SDS-polyacrylamide gel electrophoresis in 10% acrylamide gel. The protein was transferred onto a nitrocellulose membrane and blocked for 60 minutes. The membrane was then incubated with anti-ACS (Japan Ram, Tokyo, Japan) or anti-LPL (Daiichi Pure Chemicals, Tokyo, Japan) antibodies. After washing, the membrane was incubated with horseradish peroxidase-conjugated anti-rabbit secondary antibodies (The Binding Site, Birmingham, UK). Chemiluminescence of bound antibodies was detected using a kit (Amersham BioSciences, NJ, USA). Protein contents were determined by the generally established method of Lowry using BSA as a standard. This test was performed 4 times.

## 3. RESULTS

**Figure 1:**
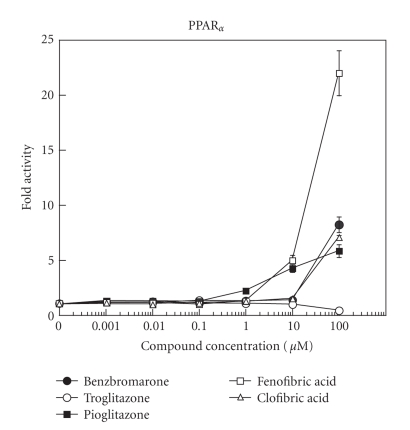
Effects of benzbromarone (•), troglitazone (∘), pioglitazone (▪), fenofibric acid (□), and clofibric acid (Δ) on PPAR_*α*_ activation. Cells were transfected with the receptor plasmid for the chimera of the PPAR_*α*_ ligand-binding domain and GAL4 DNA-binding domain, together with a reporter plasmid containing a GAL4-responsive promoter driving the expression of luciferase. Each point represents mean ± standard deviation (SD).

**Figure 2:**
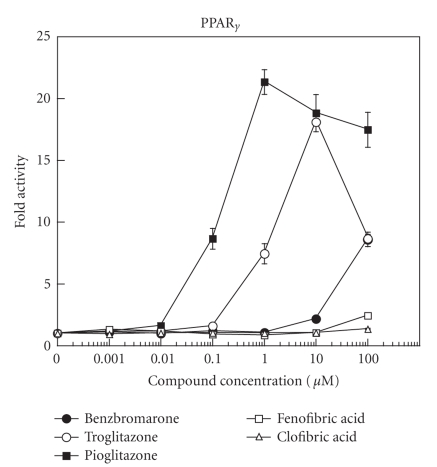
Effects of benzbromarone (•), troglitazone (∘), pioglitazone (▪), fenofibric acid (□), and clofibric acid (Δ) on PPAR_*γ*_ activation. Cells were transfected using the receptor plasmid for the chimera of the PPAR_*γ*_ ligand-binding domain and GAL4 DNA-binding domain, together with a reporter plasmid containing a GAL4-responsive promoter driving the expression of luciferase. Each point represents mean ± SD.

The binding affinity of benzbromarone for PPAR_*α*_ and PPAR_*γ*_ was examined by reporter gene assay using transcriptional activity of the PPAR target gene as a marker, in comparison with those of clofibric acid, fenofibric acid, troglitazone, and pioglitazone (Figures 1 and 2). At 100 *μ*M, benzbromarone induced an increase in luciferase activity, representing transcriptional activation of the target gene via binding of benzbromarone to PPAR_*α*_, and the magnitude was nearly equal to that induced by clofibric acid, while fenofibric acid and pioglitazone induced transcriptional activation of the target gene at concentrations of ≥10 *μ*M. Against PPAR_*γ*_, benzbromarone induced an increase in luciferase activity at a concentration of 100 *μ*M, troglitazone at ≥1 *μ*M, and pioglitazone at ≥0.1 *μ*M.

**Figure 3:**
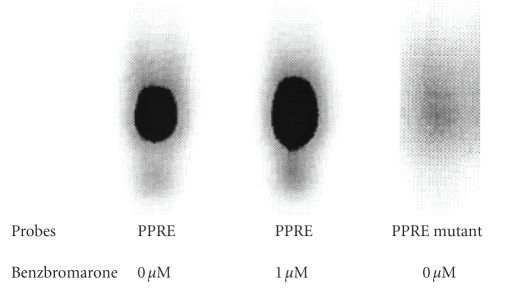
Comparative analysis of PPARs DNA-labeled PPRE complex by EMSA in nuclear extracts of 293T cells with or without benzbromarone treatment for 24 hours. Presence of PPRE was verified by incubation with mutant PPRE oligonucleotide.

After adding benzbromarone to 293T cells, the nuclear extract was incubated with fluorescence-labeled PPRE oligonucleotide, and binding activity between PPARs and PPRE was determined by EMSA. Binding between PPARs and PPRE increased in cells with benzbromarone as compared to cells without benzbromarone (Figure 3). In addition, when mutant PPRE oligonucleotide was incubated with PPARs, no evidence of binding was found.

**Figure 4:**
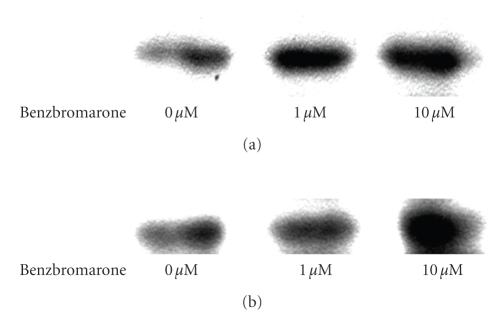
Expression levels of (a) LPL protein and (b) ACS protein as determined by Western blot analysis in 293T cells with or without benzbromarone treatment for 24 hours. The optimal density for each band measured using NIH Image. 1.6 is as follows: (a) LPL protein: 64.14 (0 *μ*M), 98.16 (1 *μ*M), 113.37 (10 *μ*M). (b) ACS protein: 51.05 (0 *μ*M), 63.29 (1 *μ*M), 94.66 (10 *μ*M).

After addition of benzbromarone to 293T cells at final concentrations of 1 *μ*M and 10 *μ*M, LPL and ACS protein expressions were determined using the western blot method, demonstrating a dose-dependent increase in each protein (Figure 4).

## 4. DISCUSSTION

PPAR regulates transcriptional activation of the target gene by forming a heterodimer with the retinoid X receptor, and recognizing and binding to PPRE via a specific gene sequence [5]. Various proteins such as LPL, ACS, fatty acid transporter, and fatty acid binding protein have been reported to be expressed as a result of transcriptional activation of target genes by PPARs [6–8].

Clofibrate and fenofibrate as PPAR_*α*_ activators and troglitazone and pioglitazone as PPAR_*γ*_ activators are considered to affect the metabolism of lipids and carbohydrates in the living body through these processes [9–11]. The present studies were performed to elucidate the details of PPAR activation by benzbromarone, which has been shown to induce hepatic hypertrophy associated with peroxisomal proliferation in rats [2].

First, the ligand affinity of benzbromarone for PPAR_*α*_ was equivalent to that of clofibric acid, a representative activator of PPAR_*α*_. The affinity of benzbromarone for PPAR_*γ*_ was weak and clearly inferior to the affinities of troglitazone and pioglitazone, which are representative activators of PPAR_*γ*_. Second, benzbromarone added to 293T cells elicited an increase in specific binding between PPARs and PPRE. Third, benzbromarone enhanced protein expression of LPL and ACS in a concentration-dependent manner.

In addition, the last experiment revealed more marked enhancement of PPAR_*α*_ expression than PPAR_*γ*_ expression by benzbromarone [4]. Moreover, based on the results of in vitro and in vivo expression analyses of various PPAR_*α*_-regulated genes in rats, benzbromarone has recently been classified as a PPAR_*α*_ agonist [12, 13]. The present findings support these reports from the perspective of intracellular gene transcription.

In patients receiving benzbromarone and in those receiving troglitazone, pioglitazone, clofibrate, fenofibrate, or other PPARs activators, hepatic impairment with an undeniable causal relationship to these drugs has been reported [14–17].

On the basis of the above findings and taking into account the mechanisms underlying increases in PPAR-mediated gene expression by PPAR_*α*_ activators such as clofibric acid and fenofibric acid and PPAR_*γ*_ activators such as troglitazone and pioglitazone, we concluded that benzbromarone can be classified as a PPAR_*α*_ activator. The hepatic hypertrophy caused by benzbromarone in rats may thus be induced via enhancement of PPAR_*α*_-mediated target gene expression and the resultant changes in lipid metabolism. In this context, the newly elucidated pharmacological features of benzbromarone could provide some hints that benzbromarone and other PPAR activators might display common mechanisms with relation to hepatic adverse effects.

The present study demonstrated that benzbromarone, a uricosuric drug, displays affinity for PPARs despite having a different chemical structure from fibrate-class antihyperlipemic drugs (fenofibrate, clofibrate) and thiazolidine-class antidiabetic drugs (troglitazone, pioglitazone), by comparison to these PPAR activators.

Considering that ${\text{C}}_{\text{max}}$ following oral administration of 100 mg benzbromarone in humans is approximately 5 *μ*M [18], which is lower than levels in the present study, the present in vitro results may be difficult to simply extrapolate to clinical use. However, further investigations must be carefully conducted using the present findings as reference, into the relationships between benzbromarone and glucose and lipid metabolism, to reconfirm the clinical value of benzbromarone.

## ABBREVIATIONS

ACS: Acyl-CoA synthetase
EMSA: Electric mobility shift assay
LPL: Lipoprotein lipase
PPAR_α_: Peroxisome proliferator-activated receptor α
PPAR_γ_: Peroxisome proliferator-activated receptor γ
PPRE: Peroxisome proliferator-responsive element
URAT1: Urate transporter 1

## References

[1] Enomoto A, Kimura H, Chairoungdua A (2002). Molecular identification of a renal urate-anion exchanger that regulates blood urate levels. *Nature*.

[2] Butler EG, Ichida T, Maruyama H, Schulte-Hermann R, Williams GM (1990). Toxicological studies on a benzofurane derivative. II. Demonstration of peroxisome proliferation in rat liver. *Toxicology and Applied Pharmacology*.

[3] Cornwell PD, De Souza AT, Ulrich RG (2004). Profiling of hepatic gene expression in rats treated with fibric acid analogs. *Mutation Research/Fundamental and Molecular Mechanisms of Mutagenesis*.

[4] Kunishima C, Inoue I, Oikawa T, Katayama S (2003). The metabolism, toxicity and pharmacological studies of benzbromarone (Urinorm). *The Journal of Saitama Medical School*.

[5] Girnun GD, Domann FE, Moore SA, Robbins MEC (2002). Identification of a functional peroxisome proliferator-activated receptor response element in the rat catalase promoter. *Molecular Endocrinology*.

[6] Schoonjans K, Peinado-Onsurbe J, Lefebvre A-M (1996). ${\text{PPAR}}_{\alpha }$ and ${\text{PPAR}}_{\gamma }$ activators direct a distinct tissue-specific transcriptional response via a PPRE in the lipoprotein lipase gene. *The EMBO Journal*.

[7] Martin G, Schoonjans K, Lefebvre A-M, Staels B, Auwerx J (1997). Coordinate regulation of the expression of the fatty acid transport protein and acyl-CoA synthetase genes by ${\text{PPAR}}_{\alpha }$ and ${\text{PPAR}}_{\gamma }$ activators. *The Journal of Biological Chemistry*.

[8] Johnson TE, Vogel R, Rutledge SJ, Rodan G, Schmidt A (1999). Thiazolidinedione effects on glucocorticoid receptor-mediated gene transcription and differentiation in osteoblastic cells. *Endocrinology*.

[9] Gebel T, Arand M, Oesch F (1992). Induction of the peroxisome proliferator activated receptor by fenofibrate in rat liver. *FEBS Letters*.

[10] Lehmann JM, Moore LB, Smith-Oliver TA, Wilkison WO, Willson TM, Kliewer SA (1995). An antidiabetic thiazolidinedione is a high affinity ligand for peroxisome proliferator-activated receptor γ (${\text{PPAR}}_{\gamma }$). *The Journal of Biological Chemistry*.

[11] Okuno A, Tamemoto H, Tobe K (1998). Troglitazone increases the number of small adipocytes without the change of white adipose tissue mass in obese Zucker rats. *The Journal of Clinical Investigation*.

[12] Kiyosawa N, Shiwaku K, Hirode M (2006). Utilization of a one-dimensional score for surveying chemical-induced changes in expression levels of multiple biomarker gene sets using a large-scale toxicogenomics database. *The Journal of Toxicological Sciences*.

[13] Tamura K, Ono A, Miyagishima T, Nagao T, Urushidani T (2006). Profiling of gene expression in rat liver and rat primary cultured hepatocytes treated with peroxisome proliferators. *The Journal of Toxicological Sciences*.

[14] Pierce EH, Chesler DL (1978). Possible association of granulomatous hepatitis with clofibrate therapy. *The New England Journal of Medicine*.

[15] van der Klauw MM, Houtman PM, Stricker BHCh, Spoelstra P (1994). Hepatic injury caused by benzbromarone. *Journal of Hepatology*.

[16] Tolman KG, Chandramouli J (2003). Hepatotoxicity of the thiazolidinediones. *Clinics in Liver Disease*.

[17] Ho C-Y, Kuo T-H, Chen T-S, Tsay S-H, Chang F-Y, Lee S-D (2004). Fenofibrate-induced acute cholestatic hepatitis. *Journal of the Chinese Medical Association*.

[18] Oikawa T, Kunishima C, Adachi Y (2004). Metabolism study of benzbromarone: in vitro metabolism and pharmacokinetics in healthy volunteers. *The Journal of New Remedies and Clinics*.

